# An Enamel Based Biopolymer Prosthesis for Dental Treatment with the Proper Bond Strength and Hardness and Biosafety

**DOI:** 10.3390/polym14030538

**Published:** 2022-01-28

**Authors:** Morakot Piemjai, Patcharee Santiwarapan

**Affiliations:** Department of Prosthodontics, Faculty of Dentistry, Chulalongkorn University, 34 Henri-Dunant Rd., Pathumwan, Bangkok 10330, Thailand; Anneemapa@gmail.com

**Keywords:** dental prosthesis biopolymer, hardness, tensile bond strength

## Abstract

Although dental prosthesis materials such as metal alloys, ceramics, and cured resin composite have long been utilized to restore teeth, their bond strength and hardness values are not well matched to human enamel. Prosthesis detachment and opposing enamel wear are major concerns in restorative dentistry. An experimental biopolymer, hybridized enamel, was synthesized and utilized as a dental prosthesis to compare hardness and tensile bond strength (TBS) with those of commercial materials. Vickers hardness (VHN) with a 100 g loading for 15 s at eight indentations on each specimen (*n* = 20) was measured. TBSs between prostheses and two types of resin luting agents (*n* = 10), Super-Bond C&B and All-Bond2 + Duo-Link, were tested. Fractured surfaces and the luting resin-prosthesis interface were examined under a stereomicroscope or a scanning electron microscope (SEM). Statistically significant differences in the TBS and hardness were revealed (*p* < 0.05). The experimental biopolymer provided a hardness value comparable with human enamel and the highest TBS for both luting agent types. The SEM micrograph demonstrated a honeycomb-like pattern interface between the experimental biopolymer and luting resin. These results suggest that this experimental biopolymer may be a better restorative material to protect from natural enamel loss from tooth reduction or attrition and prevent prosthesis detachment during mastication.

## 1. Introduction

There are four main types of materials used for fabricating dental prostheses—ceramics, metal alloys, polymers, and composites. These materials are continually being developed to obtain ideal properties for dental restorative materials. The expected requirements include biological compatibility and permanent attachment to tooth structure with physico-mechanical properties and color esthetics comparable with human enamel. Up until now, none of these materials have achieved these ideal properties [1]. Tooth-colored materials such as ceramics, porcelain fused to metal, and resin composite are widely used to make dental prostheses because of their esthetics. The major problems that contemporary materials encounter are their hardness and reduced ability to adhere to bonding or luting adhesives compared with natural human enamel. These factors affect tooth wear, abrasion or attrition, and prosthesis detachment during function which are still major factors in causing clinical failures and the short-term replacement of dental restorations [2,3].

Abrasion resistance for restorative materials should be similar to the rate of wear of tooth enamel [4]. Vickers hardness values (HV) of base metal alloys (200–395), dental porcelain (380), Lava zirconia (1250), light-cured resin composite (70–124), and human enamel (274.8 ± 18.1) have been reported [1,5,6,7,8]. Materials with a higher surface hardness than enamel tend to increase tooth wear while those with lower values are worn more easily than occluding enamel. Even though hybrid resin composites with a high content of high hardness fillers have a lower overall hardness value, they can also lead to antagonist tooth wear as well as the materials themselves [1]. However, it has been proven that wear resistance relates to hardness which is the most commonly examined mechanical property for prosthetic tooth materials [9,10].

Another important property for dental restorative or prosthesis materials is adhesion to tooth structure via bonding or luting adhesives. Complete hybridization of resin into acid-etched enamel creates a hybrid layer with cohesive failure in resin after tensile testing [11]. The hybrid layer composed of hydroxyl apatite and resin suggests mechanical adhesion is at the monomer molecular level. This high adhesion to dental enamel and less solubility in water makes resin adhesives more popularly used than acid-based cements. However, their intimate attachment, either used as the luting or bonding agent for dental prostheses or intraorally repairing restorations, respectively, mostly fail in adhesion with much less tensile bond strength (TBS) than that of tooth structure [12,13,14,15,16], which can lead to the high failure rates or short-term failure from prosthesis detachment [3]. Because of the low tensile bond strength between resin adhesive and prosthesis, more inner surface area for bonding is needed to provide the prosthesis retention against the masticatory load. Thus, more invasive tooth reduction may be required to increase the prosthesis bonding interface, which reduces the tooth strength itself.

Airborne-particle abrasion with 50 µm aluminum oxide, hydrofluoric acid etching, or chemical primer application are routinely used on the prosthesis surface to improve the bond strength to resin adhesives [13,15,16,17,18]. Hydrofluoric acid, a common conditioner to decompose glass ceramics to increase surface roughness and area for higher retention of cemented prosthesis or intraoral ceramic repairing with resin composite, can injure soft tissues after exposure. Incidents of acute and chronic symptoms after hydrofluoric acid exposure such as skin or nail burns, eye injuries, inhalation and ingestion-related symptoms, or fatality have been reviewed [19]. Therefore, it is safer for both patients and clinicians if hydrofluoric acid can be eliminated in dental restorative procedures. Exposure to bisphenol A (BPA), the main molecule of Bis-GMA which is a core for resin matrix in restorative materials or luting adhesives, has an association with the adverse effects in reproductive and developmental, metabolic disease, and other health outcomes of perinatal patients, children, and adults [20]. Thus, materials or products that are BPA-free have been developed to prevent this risk.

There are two main types of resin matrix used to fix the indirect restoration or prosthesis to tooth structure, MMA-based resin or 4-methacryloyloxyethyl trimellitate anhydride in methylmethacrylate initiated by tri-n-butylborane (4-META/MMA-TBB) resin with polymethyl methacrylate (PMMA) powder (Super-Bond C&B, Sun Medical, Shiga, Japan, C&B Metabond, Parkell Inc., Brentwood, NY, USA) and bisphenol A-glycidyl methacrylate (Bis-GMA) based resin. Tensile bond strength is the common indicator used to compare the adhesion between restorative materials and resin luting agents. Although its value depends on the surface treatments and material types, different testing methods also provide different TBS values for the same restorative material or luting resin. Direct tensile testing using mini-dumbbell shaped specimens suggests that dentin bonding via hybrid layer using 4-META/MMA-TBB resin has a significantly higher TBS than those of bonding to prosthetic materials, i.e., base metal alloy, all-ceramic, and light-cured resin composite, which have the with an average of 9–12 MPa [13]. While 4-META/MMA-TBB resin with PMMA powder can provide a very high TBS of 30–40 MPa with the PMMA resin [21].

The hypothesis of this study was that the restorative material for an indirect restoration or a prosthesis developed from biopolymer, which has a human enamel-like composition, could provide the high adhesion to resin adhesives and the proper surface hardness to reduce the tooth loss from occlusal wear or reduction to gain more retention of a prosthesis that is harmless to human health. The study objectives were to compare the microhardness and tensile bond strength of an experimental biopolymer material and the commercial products, either metal alloy, ceramics, or light-cured resin composite, when two different resin luting agents were utilized.

## 2. Materials and Methods

The research protocol was approved by the Faculty Board Committee, Faculty of Dentistry, Chulalongkorn University, Bangkok, Thailand.

### 2.1. Prosthesis Fabrication

Four types of materials were selected for testing: three commercial products, metal-based alloy (WILLIAMS, New York, NY, USA), lithium disilicate-based ceramics (IPS Empress 2, Ivoclar, Schaan, Liechtenstein), resin composite (Filtek Z250, 3M ESPE, St. Paul, MN, USA), and one experimental biopolymer. The main composition of these materials is described in Table 1. Each commercial material type, metal alloy or ceramics, was prepared using a lost wax technique in accordance with the manufacturers’ recommendations to make a 1 mm thickness inlay-like specimen with 4 mm × 5 mm inner and 5 mm × 6 mm outer surfaces using a standardized silicone mold. Experimental biopolymer specimens were shaped by a single operator. Resin composite was fully filled in the standardized silicone mold and light-cured for 40 s using Elipar Trilight (3M ESPE, USA) and finally polymerized in Labolight LVIII (GC Accord, Tokyo, Japan) for 5 min.

### 2.2. Microhardness Test

The outer surfaces of resin composite and experimental specimens were abraded with silicon carbide abrasive discs (grit #400, #600, #1000, and #1200) and polished with 0.05 µm alumina paste in wet conditions and then ultrasonically cleaned in water. Hardness measurements (*n* = 20) were conducted on glazed veneering layer of ceramics, polished outer surfaces of resin composite and experimental specimens, using a microhardness tester (Series FM-700e type D, Future-Tech, Kanagawa, Japan). The specimens were embedded in auto-polymerizing acrylic resin (Unifast, GC Dental Products Corp., Tokyo, Japan) and PMMA tubes to form a base for evaluation of the Vickers hardness number. Eight indentations (located at 4 corners with two indentations each and 0.5 mm apart) were measured on each specimen using a Vickers diamond pyramid at a 100 g indentation load for 15 s. For each specimen eight different measurements were recorded, and the results were averaged. The Vickers microhardness measurements of human enamel were made on the lingual inclined plane of lingual cusps of lower molars for a control group.

### 2.3. Tensile Bond Strength Test

**Figure 1:**
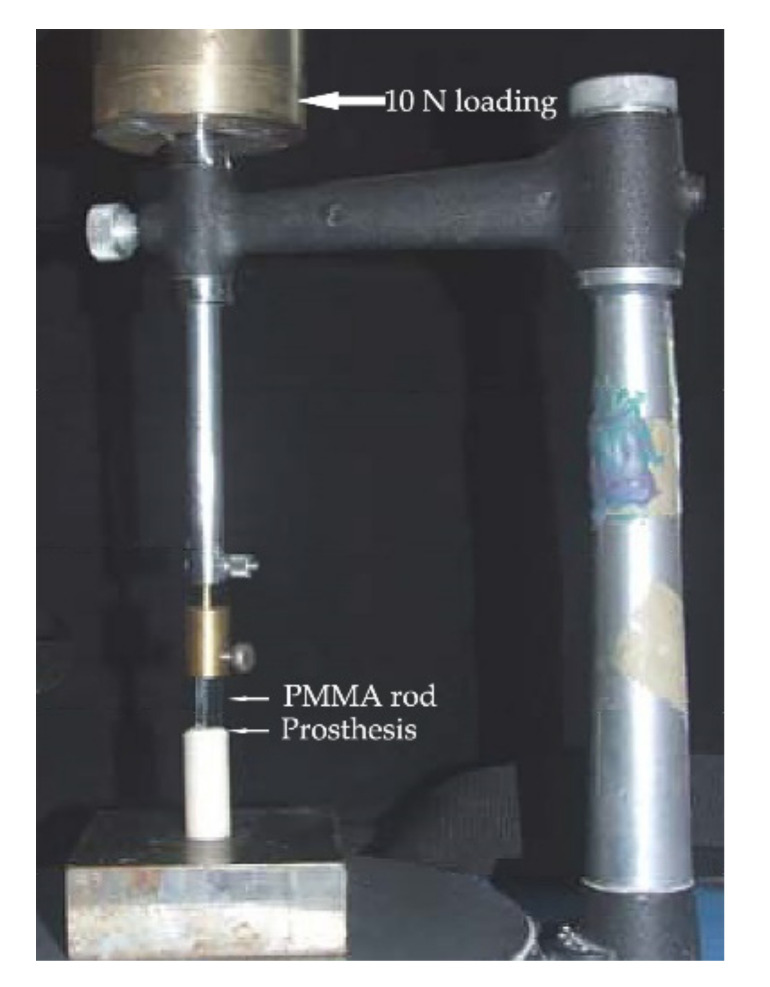
A prosthesis bonded to PMMA rod vertically aligned and loaded using a surveyor.

**Figure 2:**
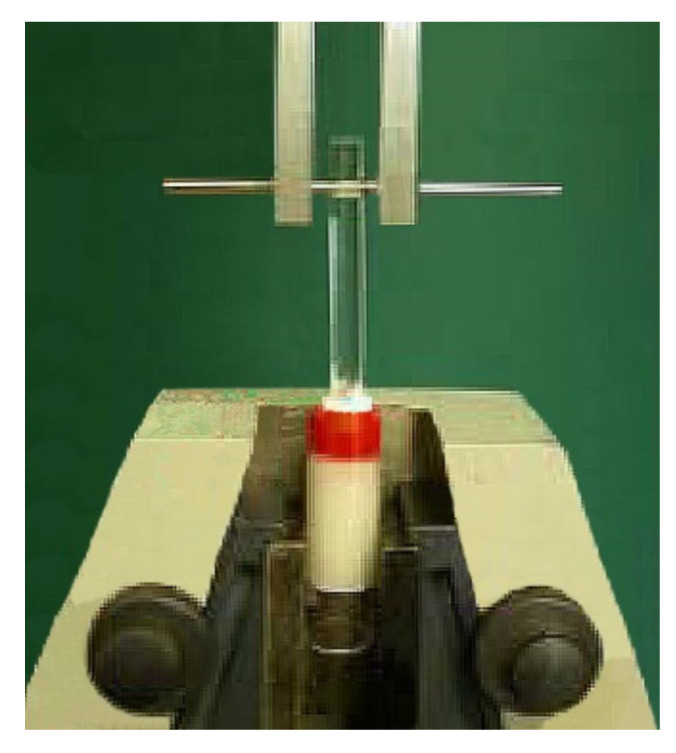
Tensile loading using a universal testing machine.

After hardness testing, the ceramic, resin composite, and experimental specimens were removed from the acrylic base to expose the inner surface for tensile testing. All inlay blocks including metal alloy, ceramic, resin composite, and experimental biopolymer were embedded in the self-cured acrylic resin in PMMA tubes with the exposed inner surface. Specimens were randomly divided into two groups of 10 specimens for each type of material to bond with two different luting agents. Top surfaces were wet abraded on silicon carbide abrasive papers (grit #400, #600) to form horizontally parallel flat surfaces. A total of 80 specimens of all groups were air-abraded with 50 µm alumina at 240 kPa pressure for 15 s at 10 mm distance and cleaned in an ultrasonic water bath for 20 min. A circular area of 3.25 mm in diameter was outlined on the prosthesis surface using one-sided adhesive tape and bonded using either 4-META/MMA-TBB or All-Bond2 + DuoLink (Bisco Inc., Schaumburg, IL, USA). resin adhesives with 5 mm diameter PMMA rod vertically aligned and loaded (10 N) using a surveyor (Figure 1). Surface treatment and bonding procedures for each material were described in Table 2. All specimens were soaked in 37 °C water for 24 h before tensile loading at a crosshead speed of 1 mm/min using a universal testing machine (Instron, Model 8872, Norwood, MA, USA) (Figure 2) following the ISO/TS 11,405 guidance [22]. The maximum forces used to pull the rods off were recorded and calculated into MPa for each group. The mode of failure was examined using a stereomicroscope (Model ML 9300, Canon, Tokyo, Japan) at 20× magnification and scanning electron microscope (SEM) at 500×, 2000× magnifications.

### 2.4. Examination of Prosthesis-Luting Resin Interface

After tensile testing, three fractured specimens from each group were randomly selected. Each experimental biopolymer specimen was vertically sectioned into two 5 mm thick specimens using a sectioning machine (IsoMet Isomet 1000 series 15, Buechler, Lake Bluff, IL, USA) for the polished and chemical challenge (soaking in 6 mol/L HCl for 30 s) specimens. The prosthesis-luting resin interface of experimental biopolymer sectioned specimens and the fractured specimens in the other groups were wet abraded with #400, #600, #1000, #1200, and #2000 grit abrasive papers and polished with 0.05 µm alumina paste. After water cleaning in an ultrasonic bath, all specimens were prepared for SEM examination from 35× to 7500× magnifications to visualize the characteristics of the interface.

## 3. Results

Means and standard deviations (SD) of microhardness values for all materials are shown in Table 3. Levene’s test disclosed inhomogeneity of variances among microhardness groups (*p* < 0.05). Brown-Forsythe and Tamhane’s multiple comparisons statistics found significant differences between groups (*p* < 0.05). The experimental biopolymer had a microhardness value close to that of enamel when compared with the other materials. A difference in microhardness value of approximately 50 VHN and 226 VHN less than that of human enamel was found in experimental and resin composite groups, respectively. While IPS Empress2 veneering ceramic provided a difference in microhardness value of approximately 214 VHN higher than that of human enamel.

The means ± SD tensile bond strength and mode of failure of the resin-prosthesis interface for all groups are summarized in Table 4. Two-way analysis of variance found significant differences in TBS values among different materials and resin adhesives as well as their interactions. Super-Bond C&B provided a significantly higher TBS than All-Bond2 + DuoLink. Brown-Forsythe and Tamhane tests revealed significant differences between groups (*p* < 0.05). The experimental biopolymer provided the highest TBS for both luting agents similar to that of resin composite. Ceramic material had the lowest TBS compared with the others when bonded with the same luting agent.

**Figure 3:**
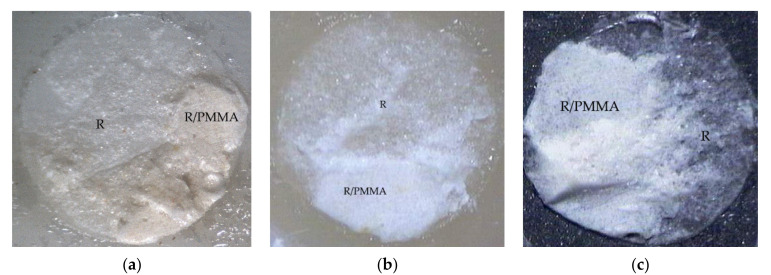
Mixed failure of cohesive in resin (R) and adhesive at resin-PMMA rod interface (R/PMMA) on fractured specimens of (**a**) experimental biopolymer, (**b**) resin composite, and (**c**) metal alloy, using Super-Bond C&B (original 20×).

**Figure 4:**
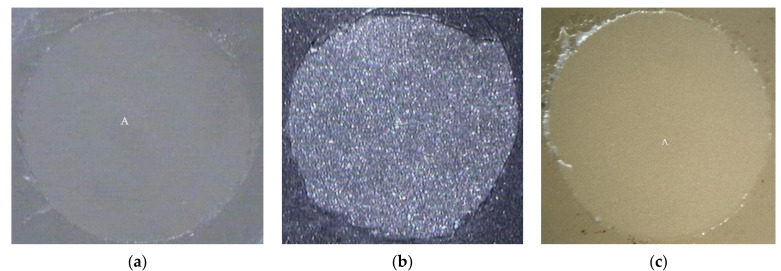
Adhesive failure on the prosthesis side interface (A) on fractured specimens of (**a**) ceramic, (**b**) metal alloy, and (**c**) resin composite using All-Bond2 + DuoLink (original 20×).

**Figure 5:**
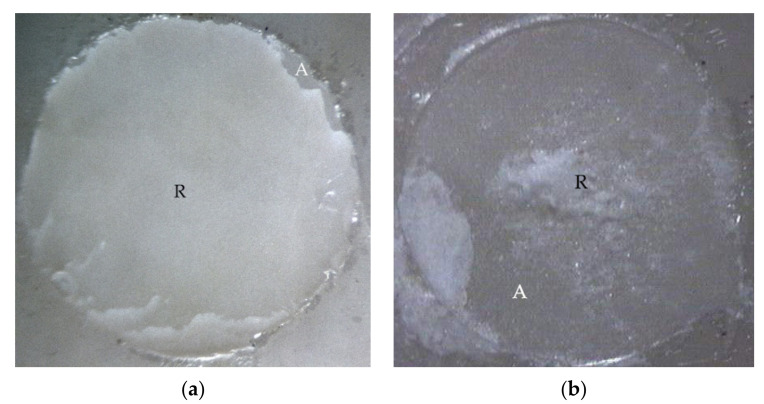
Mixed failure of cohesive failure in resin (R) and adhesive failure on the prosthesis side interface (A) on fractured specimens of (**a**) experimental biopolymer bonded with All-Bond2 + DuoLink and (**b**) ceramic bonded with Super-Bond (original 20×).

The mixed failure of cohesive failure in resin and adhesive failure at PMMA rod side interface (R, R/PMMA) was examined in fractured specimens of experimental biopolymer, metal alloy, and resin composite using Super-Bond C&B groups (Figure 3) with the highest TBS. Adhesive failure on the prosthesis side surface (A) was found in all materials (except experimental biopolymer) using All-Bond2 + DuoLink (Figure 4) with the lower and least TBS. Mixed failure of cohesive failure in resin and adhesive failure on the prosthesis side interface (R, A) was found in the experimental biopolymer bonded with All-Bond2 + DuoLink and ceramic bonded with Super-Bond groups (Figure 5).

**Figure 6:**
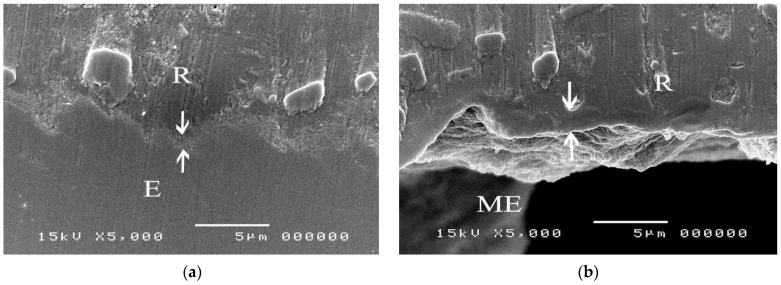
SEM micrographs of fractured experimental biopolymer bonded with Super-Bond demonstrating the consistent thickness of the hybrid layer (arrowed) at the prosthesis-luting resin interface in (**a**) polished and (**b**) chemically challenged specimens (original 5000×, E = experimental biopolymer, R = resin, ME = modified experimental biopolymer).

**Figure 7:**
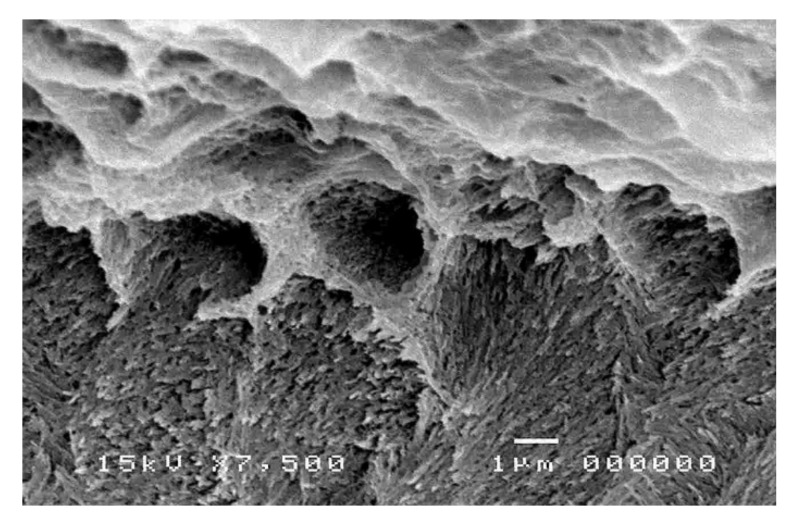
An SEM micrograph of fractured experimental biopolymer bonded with Super-Bond specimen after chemical challenge demonstrating the honeycomb-like pattern at the interfacial area (original 7500×).

**Figure 8:**
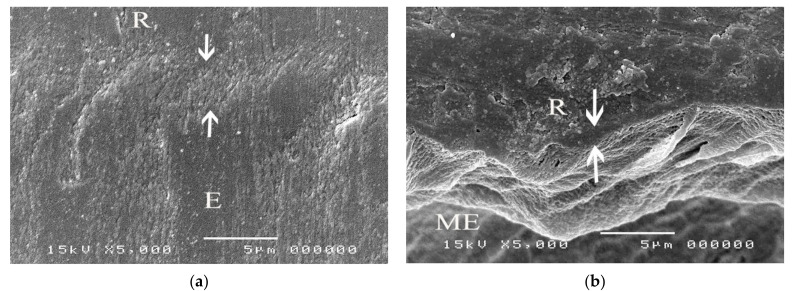
SEM micrographs of fractured ceramic bonded with Super-Bond demonstrating: (**a**) the hybrid layer (arrowed) in a polished specimen and (**b**) the thinner and degradation layer after chemical challenge (original 5000×, E = experimental biopolymer, R = resin, ME = modified experimental biopolymer).

**Figure 9:**
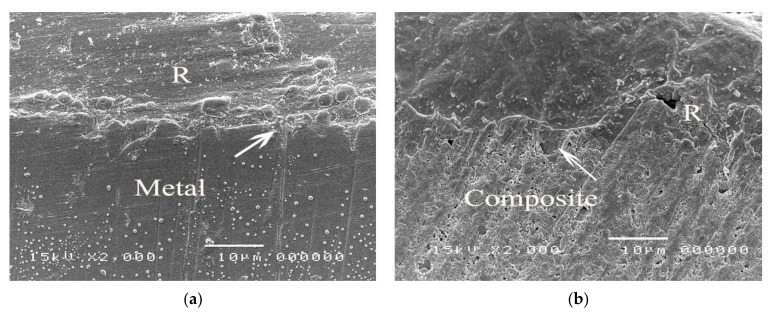
SEM micrographs demonstrating well-impregnated Super-Bond resin into the irregular pits and fissures (arrowed) of the fractured specimens in (**a**) metal alloy and (**b**) resin composite specimens (original 2000×, R = luting resin).

**Figure 10:**
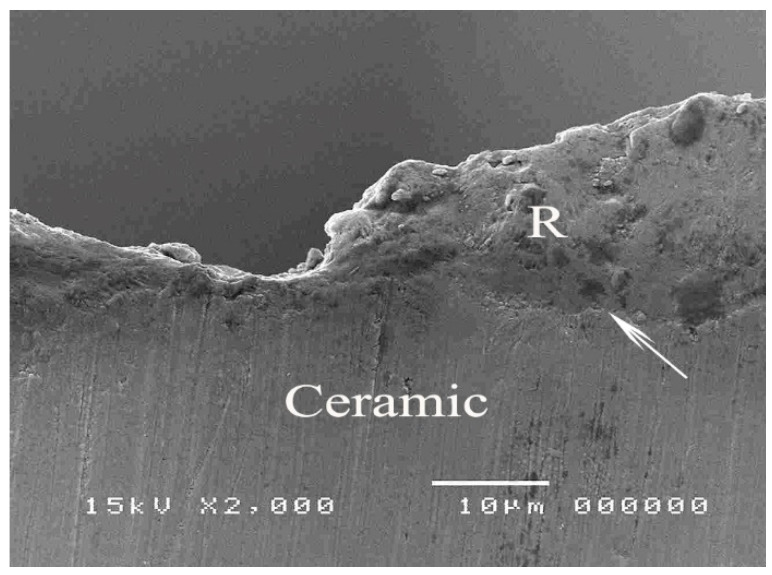
An SEM micrograph of the resin-ceramic interface using Super-Bond demonstrating the shallow irregularity on the ceramic surface of the fractured specimen (original 2000×, R = luting resin).

The SEM micrographs of experimental biopolymer and Super-Bond interfaces demonstrated the consistent thickness of the hybrid layer in the prosthesis material both before and after chemical immersion producing a honeycomb-like pattern (Figure 6 and Figure 7). The degradation of the hybrid layer after HCl immersion was found in the experimental biopolymer bonded with All-Bond2 + DuoLink (Figure 8). The interface of metal alloy and resin composite using Super Bond showed well-impregnated resin in the irregular pits and fissures formed by air-abrading with 50 µm alumina (Figure 9), whereas the bonded resin-ceramic interface using Super-Bond showed less irregularity of the ceramic surface with some area of resin detachment (Figure 10).

## 4. Discussion

Vickers hardness is widely used to assess the mechanical properties of dental restorative materials such as resin-based composites and ceramics because it is easier to use compared with other hardness tests. The hardness value has strong correlations with elastic modulus and fracture toughness values [23], which can predict the durability and wear resistance of restorations. In Table 3, the mean VHN of experimental biopolymer (287) is less than the VHN of human enamel (336); this suggests that it is rarely possible for this material to wear the human enamel. The lithium disilicate-based veneering glass ceramic provided the highest VHN (550) suggesting its greatest ability to wear opposing enamel as it has been reported in clinical studies [24,25]. The light-cured microhybrid Z250 resin composite revealed the lowest VHN (110) with 226 VHN lower than that of enamel and hence the higher wear rate reported earlier [26,27]. This may result in shorter-term replacement when occluding with ceramics or based metal alloy. The experimental biopolymer which is composed mainly of bovine enamel, and has 50 VHN less than that of human enamel could be best in terms of preventing antagonist tooth wear, wear resistance, and durability under masticatory load. The least enamel loss occurred when natural enamel occludes against natural enamel [28]. 

The tensile bond strength test is widely used to compare the adhesive properties of dental restorative materials as well as between tooth and materials. The TBS values between dental restorative materials and luting resin or bonding agents depend on the test methods [29], thus the TBS values can only be reliably compared when using the same standardized method. The results in Table 4 show the statistically significant highest values (20–22 MPa) of metal alloy, resin composite, and experimental biopolymer when bonded with Super-Bond resin. The mode of failure (R, R/PMMA) in these highest TBS groups (Figure 3) suggests that 4-META/MMA-TBB resin can penetrate well and adhere to the micro-roughened prosthesis surface created by being air-abraded with 50 µm alumina either with or without phosphoric acid conditioning better than the tensile strength of cured Super-Bond. The least TBS values (1–2 MPa) were found in metal alloy and ceramic bonded with All-Bond2 + DuoLink resin with adhesive failure (A) (Figure 4a,b). This suggests that there is the least content of Bis-GMA-based resin attached to the air-abraded surface of metal alloy and ceramics after polymerization is initiated. However, this resin could partially infiltrate more into the roughened resin composite surface and micro-porosity surface of alumina-blasted and acid-etched experimental biopolymer resulting in the higher TBS values of 9–11 MPa with adhesive failure (A) (Figure 4c) and mixed failure (R, A) (Figure 5a), respectively. The roughened surface of IPS Empress 2 ingot after air-abrasion with 50 µm alumina and bonding with Super-Bond provided an average TBS of 10 MPa with mixed failure (R, A) (Figure 5b) lower than the other materials, but higher than that bonded with All-Bond2 + DuoLink (1 MPa) with adhesive failure (A) (Figure 4a). These results suggest that type of prosthesis materials and the ability of resin monomers to penetrate micro-spaces are the main factors contributing to the high TBS.

Super-Bond C&B is the MMA-based resin that contains 4-META for a higher penetration rate of MMA and TBB which can initiate a polymerization reaction in the presence of oxygen and water. The molecular weight and viscosity of 4-META/MMA-TBB are much less than those of All-Bond2 + DuoLink, which is mainly composed of Bis-GMA, biphenyl dimethacrylate, and urethane dimethacrylate, therefore, it can effectively penetrate the micro space easier and faster before the polymerization starts. SEM micrographs of the prothesis-luting resin interface of the experimental polymer (Figure 6) demonstrated the consistent thickness of the hybrid layer (2–3 µm) after immersion in HCl solution. This acid-resistant layer with a honeycomb-like pattern at the interfacial area (Figure 7) suggests high permeability of acid-etched biopolymer for the impregnation of 4-META/MMA-TBB resin. In contrast, the degradation of the hybrid layer after soaking in HCl solution (Figure 8) suggests the partial impregnation of All-Bond2 + DuoLink, which leads to the mixed failure (R, A) with a lower TBS (Figure 5a, Table 4).

The prothesis-luting resin interface of fractured metal alloy and resin composite bonded with Super-Bond specimens (Figure 9) suggests the better ability of 4-META/MMA-TBB to penetrate in the deep pits and fissures (4–5 µm) created using air-abrasion with 50 µm alumina resulting in a higher bond strength against the polymerization contraction forces as well as higher tensile strength than that of cured Super-Bond. The interface of ceramic bonded with Super-Bond specimens after tensile loading (Figure 10) demonstrated the shallow irregular surface of ceramic resulting in the lower TBS with mixed failure (R, A) (Table 4, Figure 5b). This result implies that the IPS Empress 2 ingot surface has the highest abrasive resistance against 50 µm alumina blasting compared with the other materials. Therefore, to gain more TBS, the hydrofluoric acid application was recommended to provide micro-undercut spaces on the glass matrix for resin adhesive infiltration [30,31]. For the health concern, experimental biopolymer is composed mainly of natural bovine enamel (90%) and PMMA resin, which is BPA-free and has no need for hydrofluoric acid treatment. The results of this study support the study hypothesis.

## 5. Conclusions

A dental prosthesis fabricated using a biopolymer provided a microhardness value similar to that of human enamel and the highest TBS for both 4-MATA/MMA-TBB and All-Bond2 + DuoLink resin. With the simple and safe surface treatment, 50 µm alumina blasting and phosphoric acid etching, the experimental biopolymer surface could gain a TBS higher than the tensile strength of cured Super-Bond C&B. According to the best properties compared with the other tooth-colored tested materials, this enamel-based biopolymer may be the first choice for a dental prosthesis fabricated using CAD/CAM (Computer-aided design/Computer-aided manufacturing) technology.

## Figures and Tables

**Table 1:** Main composition of the tested materials.

Type of Materials	Main Composition
Metal alloy	Ni-Cr-Be-based alloy
Ceramics	Lithium disilicate in glass matrix core ingot, Veneering Layer, Glaze liquid
Resin composite	Bis-GMA (Bisphenol A diglycidyl ether dimethacrylate), UDMA (urethane dimethacrylate), Bis-EMA (Bisphenol A polyethylene glycol diether dimethacrylate), 0.01–3.5 µm silica/zirconia fillers (60% by volume)
Experimental Biopolymer	Etched bovine enamel infiltrated with methylmethacrylate resin

**Table 2:** Manipulation of prosthesis surface conditioning and adhesive resin.

Procedures	Materials Type	Super Bond C&B	All-Bond2 + Duo-Link
Surface treatment	Experimental biopolymer	Conditioned with 65% H_3_PO_4_ for 30 s, rinsed off for 10 s, air-dried for 10 s	Conditioned with 32% H_3_PO_4_ for 15 s, rinsed off for 15 s, air-dried,
Bonding Procedure	Experimental biopolymer, Ceramic, Resin composite	Applied Porcelain liner M with a sponge, air-dried 5 s Applied 4-META/MMA-TBB and PMMA powder using brush-dip technique	Applied primer 5 times, gently air-dried 5 s, applied thin layer of D&E resin, and DuoLink cement, light-cured for 40 s
	Metal alloy	Applied 4-META/MMA-TBB and PMMA powder using brush-dip technique	Applied primer twice, gently air-dried 5 s, applied pre-bond resin, gently air-dried, and applied DuoLink cement, light-cured for 40 s

**Table 3:** Mean ± SD of microhardness value (Vickers hardness number; VHN) for all groups.

Group (*n* = 20)	Vickers Hardness Number
Ceramic	550.02 ± 7.90
Resin composite	109.79 ± 3.31
Experimental biopolymer	287.16 ± 6.42
Enamel	336.12 ± 11.65

Significant differences were found between each group at *p* < 0.05.

**Table 4:** Mean ± SD of tensile bond strength (MPa) and failure modes for all groups (*n* = 10).

	Tensile Bond Strength (Failure Modes)	
Materials	Super-Bond C&B	All-Bond2 + Duo-Link
Experimental biopolymer	20.45 ± 5.21 ^a^ (R, R/PMMA)	11.95 ± 2.85 ^b^ (R, A)
Metal alloy	22.00 ± 2.93 ^a^ (R, R/PMMA)	2.12 ± 0.77 ^c^ (A)
Ceramic	10.49 ± 1.40 ^b^ (R, A)	1.38 ± 0.41 ^c^ (A)
Resin composite	20.38 ± 3.66 ^a^ (R, R/PMMA)	9.67 ± 2.27 ^b^ (A)

^a,b,c^ Statistically significant differences between groups shown with different superscripts (*p* < 0.05). A = Adhesive failure at prosthesis side interface, R = Cohesive failure in the cured resin, R/PMMA = Adhesive failure at PMMA rod side interface.

## Data Availability

The data presented in this study are available on request from the corresponding author.
